# A European framework for the assessment of digital health technologies: conceptual advances, challenges, and future directions

**DOI:** 10.3389/fdgth.2026.1809237

**Published:** 2026-06-17

**Authors:** Chiara de Waure, Ilaria Valentini, Nicolò Scarsi, Wija Oortwijn, Zoe Garrett, Iga Lipska, Rossella Di Bidino, Emmanouil Tsiasiotis, Americo Cicchetti, Dario Sacchini

**Affiliations:** 1 Department of Medicine and Surgery, University of Perugia, Perugia, Italy; 2 The Graduate School of Health Economics and Management (ALTEMS), Università Cattolica del Sacro Cuore, Rome, Italy; 3 Section of Hygiene, Department of Life Sciences and Public Health, Università Cattolica del Sacro Cuore, Rome, Italy; 4 IQ Health Science Department, Radboud University Medical Center, Nijmegen, Netherlands; 5 National Institute for Health and Care Excellence, Manchester, United Kingdom; 6 Health Policy Institute, Gdańsk, Poland; 7 Center for Competence Development, Integrated Care and e-Health, Medical University of Gdańsk, Gdańsk, Poland; 8 Fondazione Policlinico Universitario Agostino Gemelli IRCCS, Università Cattolica del Sacro Cuore, Rome, Italy; 9 Research Centre for Clinical Bioethics and Medical Humanities (CRiBCeMH), Università Cattolica del Sacro Cuore, Rome, Italy

**Keywords:** digital health implementation, digital health technologies, EDiHTA framework, EU-funded project, health technology assessment

## Abstract

Digital health technologies (DHTs) are transforming healthcare by enabling for example new care models, improving patient access and engagement, and streamlining healthcare workflows. They have the potential to increase efficiency, reduce administrative burdens, and enhance the overall quality of care. However, across Europe, DHTs evaluation frameworks remain fragmented, and while new approaches continue to emerge, they often focus on specific types of DHTs or particular stages of the product lifecycle. Moreover, traditional Health Technology Assessment (HTA) frameworks have been developed mostly to evaluate medicinal products, which may limit their applicability to DHTs, ultimately constraining health systems’ ability to capture DHTs’ added value. The European Digital Health Technology Assessment (EDiHTA) is a EU-funded project, which started in January 2024 to co-create an HTA framework for DHTs in order to face these challenges. It is aimed at co-developing together with different key stakeholders a comprehensive and flexible assessment framework tailored to different DHTs, applicable across DHTs lifecycle and across EU member states. In this perspective article, we present our concept design that is being used as foundation of the development of the EDiHTA framework, and we address current challenges in the assessment of DHTs, and the opportunities that lie ahead.

## 1 Introduction

Over the past decade, digital health has evolved from experimental innovations to integral components of health systems worldwide (1). The World Health Organization (WHO) defines digital health as the development and use of digital technologies to support health system functioning and improve health outcomes. This means that digital health makes use of tools and services relying on information and communication technologies that encompass, among others, eHealth [which includes mobile health (mHealth)], telehealth, and emerging technologies (2, 3).

Therefore, digital health technologies (DHTs) is an umbrella term that includes this variety of technologies, which are used by diverse groups, including the public, patients and caregivers, healthcare professionals, and health system managers (4–6). Today, some DHTs such as mHealth applications, wearable sensors, remote monitoring platforms, and telemedicine services are widely implemented, while AI-based tools are increasingly integrated into diagnostic processes and clinical decision-making pathways (7–9). Collectively, these technologies hold the potential to increase efficiency and clinical, economic and organizational effectiveness, strengthen patient empowerment, and foster new models of care delivery, thereby transforming health systems (10). However, recent reviews have underscored a range of organizational challenges, including gaps in evidence generation, insufficient stakeholder engagement, limited infrastructure readiness, and inadequate workforce training, that complicate the implementation of DHTs across diverse healthcare settings (11).

As such, evaluating DHTs remains a challenging task. Health Technology Assessment (HTA) is defined as a “multidisciplinary process that uses explicit methods to determine the value of a health technology at different points in its lifecycle; the purpose is to inform decision-making in order to promote an equitable, efficient, and high-quality health system” (12). Traditional HTA frameworks, such as the European network for HTA (EUnetHTA) Core Model (13), have been designed for assessing mostly drugs but also medical devices, and could be ill-suited for DHTs too (14). Nonetheless, DHTs evolve continuously, often incorporate AI, have broad intended uses and may shift purpose in the post-deployment stage. Therefore, some countries have developed dedicated HTA frameworks for DHTs assessment to inform decisions regarding their implementation, such as Spain (15), Finland (16) and England (17). As DHTs are increasingly deployed across countries, a supranational framework is essential to harmonize their assessment, which is comprehensive but at the same time flexible to address the needs of different stakeholders and cover the whole DHTs lifecycle, while remaining applicable across decision-making levels (EU, national, regional, and local).

In response to this, the European Digital Health Technology Assessment (EDiHTA) project is a 4-year Horizon Europe Research and Innovation Action funded under call HORIZON-HLTH-2023-IND-06-07 and launched in January 2024 (18). It brings together more than 15 partners from European zone countries (19) to co-create the first European digitalized HTA framework, which is being co-designed with input from key stakeholders, tested for validity, and piloted for practical implementation. Its purpose is to harmonize the assessment of DHTs at EU level, while ensuring applicability across all categories of DHTs and at every life cycle stage according to the Healthcare Innovation Cycle (19).

## 2 Conceptualizing the EDiHTA framework via co-creation

One of the strengths of the EDiHTA framework lies in the way it was conceptualized and developed. Rather than being designed behind closed doors by a small group of experts or academics, it emerged through an open and iterative co-creation process with key stakeholders as outlined in the Roadmap to Innovation of HTA Methods (IHTAM) (20). The process included mapping and critically appraising existing HTA frameworks for DHTs, and cross-checking their assessment requirements with those emphasized by key stakeholders. From the mapping it emerged that two frameworks are currently used to assess DHTs, namely the EUnetHTA Core Model and AQuAS (Agency of Health Quality and Assessment of Catalonia) framework. This second one provides one of the first structured approaches to DHTs evaluation but it remains confined to the local context in which they were developed or to not a specific stage of a DHT lifecycle (15). The views of stakeholders were collected via semi-structured interviews and focus group, facilitating multi-stakeholder workshops, and validating preliminary ideas through targeted surveys. The focus group included 29 DHT developers and manufacturers. In addition, interviews and focus groups were conducted with 22 healthcare providers, 16 policy makers and regulators, 15 patients and patient organizations in collaboration with the European Patient Forum, across 10 European countries in 2024 (21). Crucially, the process engaged a wide spectrum of stakeholders, namely policymakers, HTA agencies, clinicians, patients, technology developers, academics, and healthcare providers, ensuring that the resulting framework would not only be methodologically sound but also legitimate and acceptable across different decision-making contexts.

This participatory approach enhanced the framework's robustness by embedding practical perspectives from those who will ultimately use or be affected by it. It also created a sense of ownership among stakeholders, which is fundamental for achieving real adoption and avoiding the perception of the model as a purely academic or top-down exercise.

The final concept design encompasses 13 assessment domains. In Table 1 we present the comparison of the domains of the EDiHTA concept design with those of two reference models, namely the EUnetHTA Core Model and the AQuAS framework. EUnetHTA was chosen as the benchmarking model given its broad recognition and widespread uptake in HTA while the AQuAS framework was considered because it served as reference for extracting information and data on DHT-related HTA methodological guidance and HTA reports within the EDiHTA project.

**Table 1:** Comparison of assessment domains across the AQuAS framework, EUnetHTA core model, and EDiHTA concept design. For AQuAS and EUnetHTA, we reported domain titles only (as per the original frameworks); domain descriptions were provided for the EDiHTA concept design.

AQuAS framework (15)	EUnetHTA Core Model (13)	EDiHTA concept design domains	EDiHTA concept design domains definition
Description of Health problem and Current Use of the Technology	Health problem and current use of technology	Health problem and current management	Description of the health problem including epidemiology (prevalence, incidence), disease burden on individuals and society, physiopathology, natural history, and the target population among which the digital health technology is applied or expected to be applied. It reports also the information regarding the current management of the condition and the specific context to which it is applied.
DHT specific aspects	Partly under Description and technical characteristics	Description of the technology	Description of the digital health technology characteristics (e.g., design, operational features, evidence-based content, material requirements, needed training), including the context and conditions in which it is introduced, the purposes for which it is intended, the level of care at which it is applied and the regulatory status (certifications or licenses, compliance with recognized standards).
		Digital aspects	Evaluation of the technological elements and functions that shape and characterize the digital health technology, including usability, interoperability, adaptability, reliability, technical stability, algorithmic performance metrics, generic reproducibility, interpretability, and transparency to ensure that algorithmic processes are explainable and free from bias, and manageability and compatibility, including evaluations of system monitoring processes and error handling.
Safety	Safety	Safety	Evaluation of risks, and unwanted, undesired or harmful effects arising by using digital health technology. This includes physical and psychological risks (clinical safety) as well as risks in terms of privacy or quality of information (technical safety).
Clinical efficiency and effectiveness	Clinical effectiveness	Clinical efficacy and effectiveness	Evaluation of the clinical benefits and the impact on health status and quality of life of the digital health technology, compared to standard or alternative interventions, under controlled conditions (i.e., ideal circumstances for assessing efficacy), or uncontrolled conditions (i.e., under usual health care practice for assessing effectiveness).
Economic aspects	Cost and economic evaluation	Economic aspects	Evaluation of the costs (costs of acquisition, maintenance and use both at the patient/user and health system level) of the digital health technology and economic evaluation of it compared to existing alternatives.
Organisational aspects	Organisational aspects	Organisational aspects	Evaluation of the organisational impact of the digital health technology and of the infrastructure and resources needed to be mobilized and organized to implement the technology (e.g., human resources and workload, skills and knowledge, training, work culture, attitudes and material artefacts).
Ethical aspects	Ethical analysis	Ethical aspects	Evaluation of the ethical concerns of the digital health technology, i.e., prevalent social and moral norms and values that the digital health technology itself constructs and influences in the (socio-political, cultural, legal, religious, and economic) context in which it is implemented or intended to be used.
Patient aspects	Patient and social aspects	Patients-related aspects	Evaluation of patients’, healthy individuals’ and caregivers’ perspectives that may have an impact on the use of the digital health technology (i.e., experiences, attitudes, preferences, values, and expectations, such as acceptability to use the digital health technology, ease of using the digital health technology, digital health literacy, commitment/adherence to the digital health technology, perceived benefit the digital health technology)
Sociocultural aspects	Patient and social aspects	Sociocultural aspects	Evaluation of sociocultural impact that the digital health technology may have (e.g., accessibility to the service or health care, changes in workflows and roles, modification in the doctor-patient relationship, etc.) with respect to specific groupings of patients or individuals, such as older people, people living in remote communities, people with learning disabilities, ethnic minorities, immigrants etc.
Environmental aspects	-	Environmental aspects	Evaluation of the direct and indirect environmental impact associated with the development and implementation, use and disposal of digital health technology.
Data privacy, security, EU regulation	Partly under Legal aspects	Legal and regulatory aspects	Evaluation of the degree to which digital health technology complies with the regulations, rules and standards of the country/region in which it is planned to be implemented.
Post deployment monitoring	Partly under Organisational aspects	Post deployment monitoring aspects	Description of the mechanisms established for post-deployment assessment of the digital health technology by its developers and/or those responsible for its management.

Some domains were confirmed without any substantial change, such as health problem description, safety, clinical efficacy and effectiveness, economic and organizational aspects, ethical and sociocultural aspects. On the contrary, the rest of domains have been specifically operationalized for DHTs to capture issues that are particularly salient in this context. These include description of the technology, digital aspects, patients-related aspects, environmental impact, legal and regulatory aspects and post-deployment monitoring (see Table 1).

The primary challenges encountered during the conceptual design phase were the integration of diverse stakeholder requirements and the mitigation of potential overlaps between assessment domains. To address the former, the consortium expanded upon the AQuAS framework by introducing a dedicated “digital aspects” domain. To resolve the latter, precise definitions for each domain were developed based on existing frameworks and subsequently finalized through a formal consortium consensus.

## 3 Taxonomy and contextualization

In addition to assessment domains and their definitions, it is important to have a unified taxonomy to classify DHTs in order to determine the evidence requirements across their lifecycle. DHTs are not alike: a wellness app designed to track physical activity is profoundly different from an AI-driven insulin pump capable of autonomously adjusting dosage. As part of the EDiHTA project, a taxonomy for DHTs has been developed using a literature review, a survey of HTA agencies, and a modified Delphi study with 82 experts in the field (22). The EDiHTA concept design includes the following three taxonomy criteria that align with the applicable regulations such as the MDR 2017/745 (23) and the AI act (24):

1. *Intended purpose of the technology*: DHTs can be classified into four broad class (25): (1) DHT with no direct patient, health or care outcome intended to save cost or release staff time; (2) DHTs intended to help citizens and patients to manage their own health and wellness; (3) DHTs intended to provide general information and provide health professional training material or tools; (4) DHTs with direct health outcomes intended to treat or diagnose a specific condition or guide treatment, diagnosis and care choices. Therefore, these categories range from tools without direct patient outcomes (e.g., administrative software, workflow optimization tools) to technologies with direct clinical impact (e.g., diagnostic or therapeutic applications).
2. *AI capability*: This criterion is considered essential to capture DHTs incorporating AI at different levels of autonomy and personalization. This focuses on the level of human intervention required for a DHT to function, as well as the ability of a DHT to personalize responses to the user.
3. *Maturity level*: Identifies the DHT's maturity stage using the Healthcare Innovation Cycle Framework (26), which builds on the established Technology Readiness Level (TRL) approach (27) and adapts it to the specific challenges of healthcare innovation. It has 10 maturity levels between the steps from defining an unmet clinical need to adoption as a standard of care. Accordingly, DHTs are positioned along a continuum from early-stage concepts and prototypes (i.e., proof of concept, feasibility pilots) to validated solutions that are implemented in routine clinical practice (standard of care). For operational clarity, the consortium proposes a new reclassification of the 10 levels into four maturity classes: (i) Research and concept design (levels 1–3), (ii) Pilot (levels 4–7), (iii) Market approval (level 8), and (iv) Post-market (levels 9–10). This dimension ensures that the framework is calibrated according to the stage of development, guaranteeing its lifecycle orientation.

Together, these criteria allow for a contextualized assessment approach. This ensures that whether evaluating a wellness app, an AI chatbot for stress management, or an AI-based insulin pump, the assessment is tailored to answer the critical questions that arise throughout the technology's entire lifecycle. This layered approach reflects a fundamental truth: context matters. By tailoring evidence requirements to intended purpose, AI capability, and maturity, the framework will balance rigor with feasibility/flexibility while avoiding both under or over burdening evaluations. During the final development of the concept design a survey of consortium partners was also conducted to assess the perceived relevance of each assessment domain in relation to the intended purpose and AI capabilities of DHTs. Maturity level was not considered in this exercise because the consortium agreed to further develop and operationalize how to shape the framework in respect to this criterion during the pilot phase of the final toolkit.

**Figure 1:**
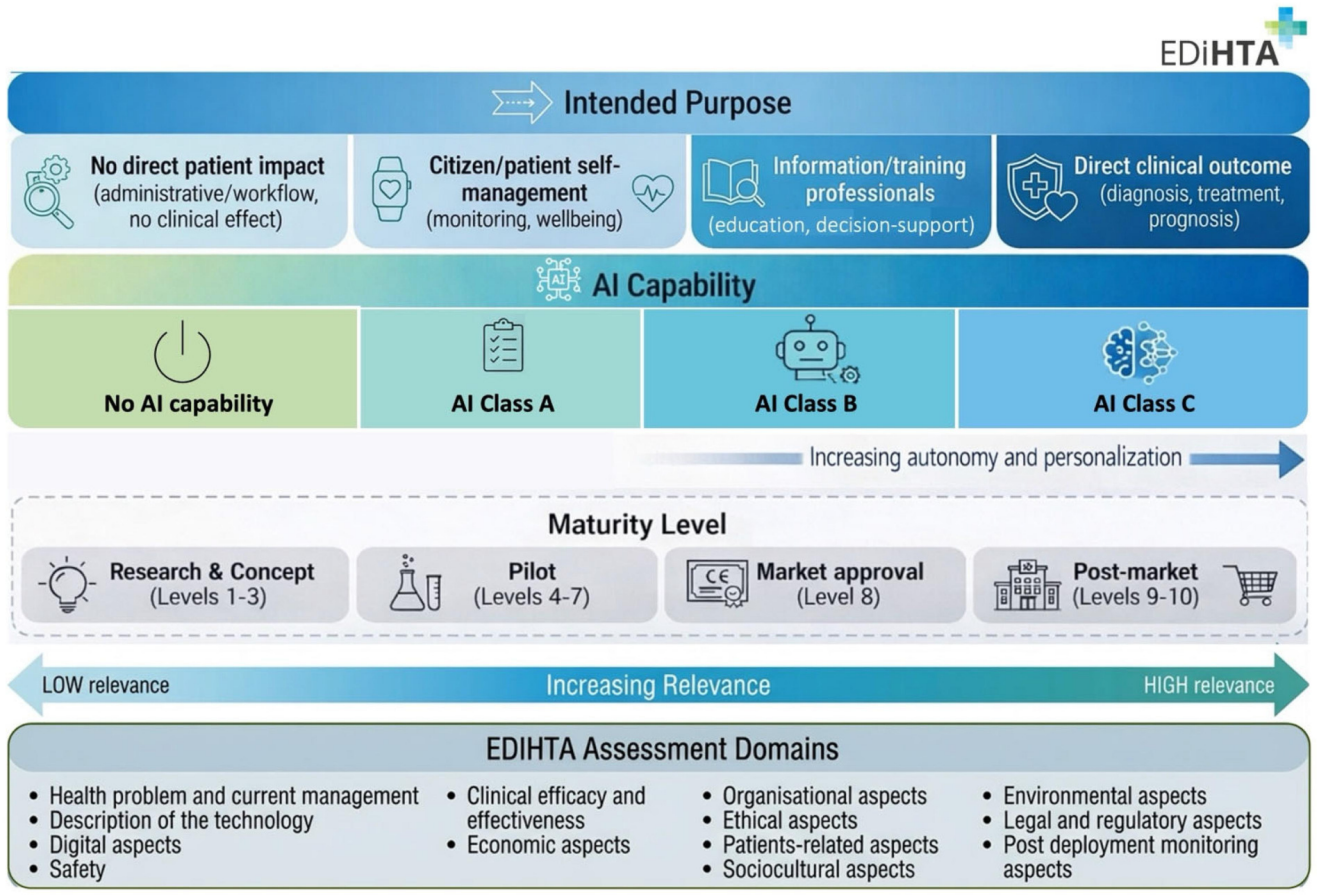
EDiHTA concept design and its contextualization to DHTs taxonomy.

Figure 1 reports the results of the survey, which indicate that the relevance of the assessment domains increases with higher levels of AI capability and depends on the intended purpose of DHT.

Specifically, consortium partners overall attributed more relevance to domains such as digital aspects, safety, legal and regulatory aspects when DHTs have more AI capabilities. When considering categories of intended purpose, environmental aspects consistently ranked low. For DHTs with direct clinical outcomes, almost all domains were considered highly relevant. The results of the survey reinforced the role of intended purpose and AI capabilities classification in guiding the assessments. A challenge faced during the discussion of the results was linked to the heterogeneity of DHTs within each identified classes of AI capability, coupled with the small number of participants. These factors limit the generalizability of the results of the survey but represents preliminary evidence to set the piloting phase of the EDiHTA framework.

## 4 Discussion

The concept design of the EDiHTA framework represents a significant advancement in adapting HTA to the realities of digital innovation. For the first time, a EU and co-developed HTA framework is being proposed to comprehensively assess any type of DHT considering a lifecycle approach. This framework is expected to mark an important milestone, signalling a transition toward a more comprehensive, flexible and forward-looking approach capable of addressing the multifaceted nature of digital transformation in healthcare.

However, this ambitious vision also raises challenges that require careful management. The inclusion of 13 domains, while ensuring comprehensiveness, risks overwhelming the assessment process. Without prioritization mechanisms, evaluations may become overly complex or resource-intensive, limiting their feasibility/flexibility in practice. Piloting the framework in real-world contexts will therefore be a crucial next step to identify strategies for balancing breadth with practicality, ensuring that assessments remain both rigorous and implementable.

Another challenge is linked to the evolving and dynamic features of DHTs. Issues such as explainability, algorithmic bias, transparency, and autonomy continue to advance rapidly, necessitating new methodologies that are adaptable rather than static. Assessment tools that fail to evolve risk becoming obsolete in the face of accelerating technological change. For this reason, the framework must embed flexible mechanisms that enable continuous updating and refinement of assessment methods.

Stakeholders heterogeneity may further complicate the evaluation process. Patients frequently point to issues such as usability and inclusiveness, whereas developers tend to stress scalability, technical aspects and broader adoption. Regulators, on the other hand, usually give precedence to safety and compliance (28). Reconciling these divergent perspectives within a unified evaluative structure is inherently complex, yet essential to ensure legitimacy, stakeholder trust, and real-world adoption.

In addition, some gaps remain. Many components of the concept design are based on conceptual consensus rather than empirical validation, meaning that its robustness can only be demonstrated through real-world application. To strengthen the first draft before implementation, consensus-building activities are planned for 2026, including a multi-stakeholder workshop and a pilot phase involving all relevant stakeholder groups. The pilot phase will follow the digitalization of the framework and the development of the user guidance.

The pilot phase will involve DHT developers, hospitals, as well as HTA agencies. A diversity of developers will be engaged to identify DHTs that will be assessed through the application of the framework. This will ensure that the framework is tested and validated across a broad spectrum of DHTs by relevant stakeholders, namely hospitals and HTA agencies. The objective of the pilot phase is twofold: first, to evaluate the framework usability and clarity, as well as its added value, from the perspective of stakeholders in real-world settings; and, second, to ensure the framework's comprehensiveness by identifying gaps and ambiguities requiring refinement prior to the final implementation of the EDiHTA digital platform.

This stepwise approach, moving from concept design to operationalization and piloting will allow iterative refinement and ensure that the framework evolves from a conceptual model into a practical and implementable assessment tool. Ultimately, the aim is to develop a harmonised European framework to assess DHTs that is flexible, lifecycle-oriented, and adaptable to the rapid evolution of digital health.

The potential benefits of a harmonized European framework are substantial. A shared framework could reduce duplication of effort, improve comparability across jurisdictions, and streamline market access procedures, ultimately accelerating patient access to safe and effective innovations. This aligns with the current regulation on HTA (29) and Medical Device Regulation (MDR 2017/745) and also supports the EU competitiveness. Moreover, by aligning with further broader EU policy initiatives such as the Artificial Intelligence Act (AI Act, Regulation 2024/1689) (24) and the European Health Data Space (30), the framework is situated within a wider regulatory and policy ecosystem. This alignment enhances coherence and strengthens governance, ensuring that DHTs are evaluated in ways that are both scientifically rigorous and politically consistent with EU policies and strategies.

The concept design of the EDiHTA framework is more than a methodological innovation; it constitutes a political and cultural shift. By involving stakeholders across Europe in its co-creation process and building on existing assessment frameworks and the understanding of DHTs complexity, EDiHTA aims to deliver a comprehensive framework that embeds different perspectives, needs and requirements.

Based on the concept design the framework is now going to be further developed to be modular and adaptable, reflecting the principle that, just as DHTs evolve continuously, the approaches used to evaluate them remain dynamic and responsive to change.

The digitalization of the framework and following pilot phase envisioned by the EDiHTA project will show its added value as it will reveal how the framework can be applied in practice without placing excessive burdens on evaluators, reflecting at the same time the complexity of DHTs and providing different policymakers with timely and relevant evidence.

## Data Availability

The data analyzed in this study is subject to the following licenses/restrictions: The original contributions presented in this study are included in the article. Requests to access these datasets should be directed to the corresponding author.
